# Somatosensory Evoked Potentials as a Tool to Evaluate Brainstem Herniation in the Neuroscience Intensive Care Unit

**DOI:** 10.7759/cureus.2443

**Published:** 2018-04-06

**Authors:** Nakul Katyal, Christopher R Newey, Pravin George, Premkumar Nattanamai, Jonathan M Beary, Agnieszka Ardelt, Anantha Vellipuram

**Affiliations:** 1 Department of Neurology, University of Missouri, Columbia, USA; 2 Neurology, Cleveland Clinic Ohio; 3 Neurobehavioral Sciences, A. T. Still University; 4 Neurology, Metro Health Cleveland; 5 Neurology, Texas Tech University Health Sciences Center Paul L Foster School of Medicine

**Keywords:** somatosensory evoked potentials (sseps), posterior fossa, tonsillar herniation

## Abstract

Somatosensory evoked potentials (SSEPs) are a sensitive, minimally invasive technique used to localize dysfunction of myelinated peripheral and central axons in the nervous system. The utility of SSEPs in acutely assessing central nervous system function in brainstem herniation in the neuroscience intensive care unit (NICU) has not been well established. We discuss a case of an 18-year-old, postpartum female who presented with intermittent headache, diplopia, nausea/vomiting and cachexia following delivery two months prior. Shortly after arrival to the emergency department, she developed flaccid quadriparesis and complete ophthalmoplegia. Computed tomography (CT) of the head showed effacement of the basal cisterns along with 2 cm cerebellar tonsillar herniation into the foramen magnum concerning for intracranial hypotension. Raising the head of bed caused hemodynamic instability necessitating prolonged Trendelenburg positioning. The patient was evaluated with serial SSEPs which initially showed a bilateral low amplitude N20 response and normal N13 response. Subsequent SSEP testing showed increased N20 amplitude which correlated with clinical improvement in the patient. SSEP is a minimally invasive and sensitive method used to assess the integrity of the somatosensory nervous system pathway; SSEPs may be a useful monitoring adjunct to assess the evolution of posterior fossa lesions leading to brainstem compression.

## Introduction

Since its first description, somatosensory evoked potentials (SSEPs) have been used as an effective means of evaluating the integrity of the somatosensory pathways [1]. SSEP monitoring is frequently used as an early indicator of intraoperative spinal cord injury [2]. SSEPs are also utilized as a prognostic tool in coma [3]. Absence of bilateral median N20 responses in postanoxic coma is highly predictive of poor clinical outcome [4]. The role of SSEPs in evaluating posterior fossa lesions in the neuroscience intensive care unit (NICU) has not been well established. We present a case utilizing SSEPs as a neuro-monitoring tool demonstrating a correlation between electrical improvement and clinical recovery in a patient with intracranial hypotension and tonsillar herniation.

## Case presentation

**Figure 1:**
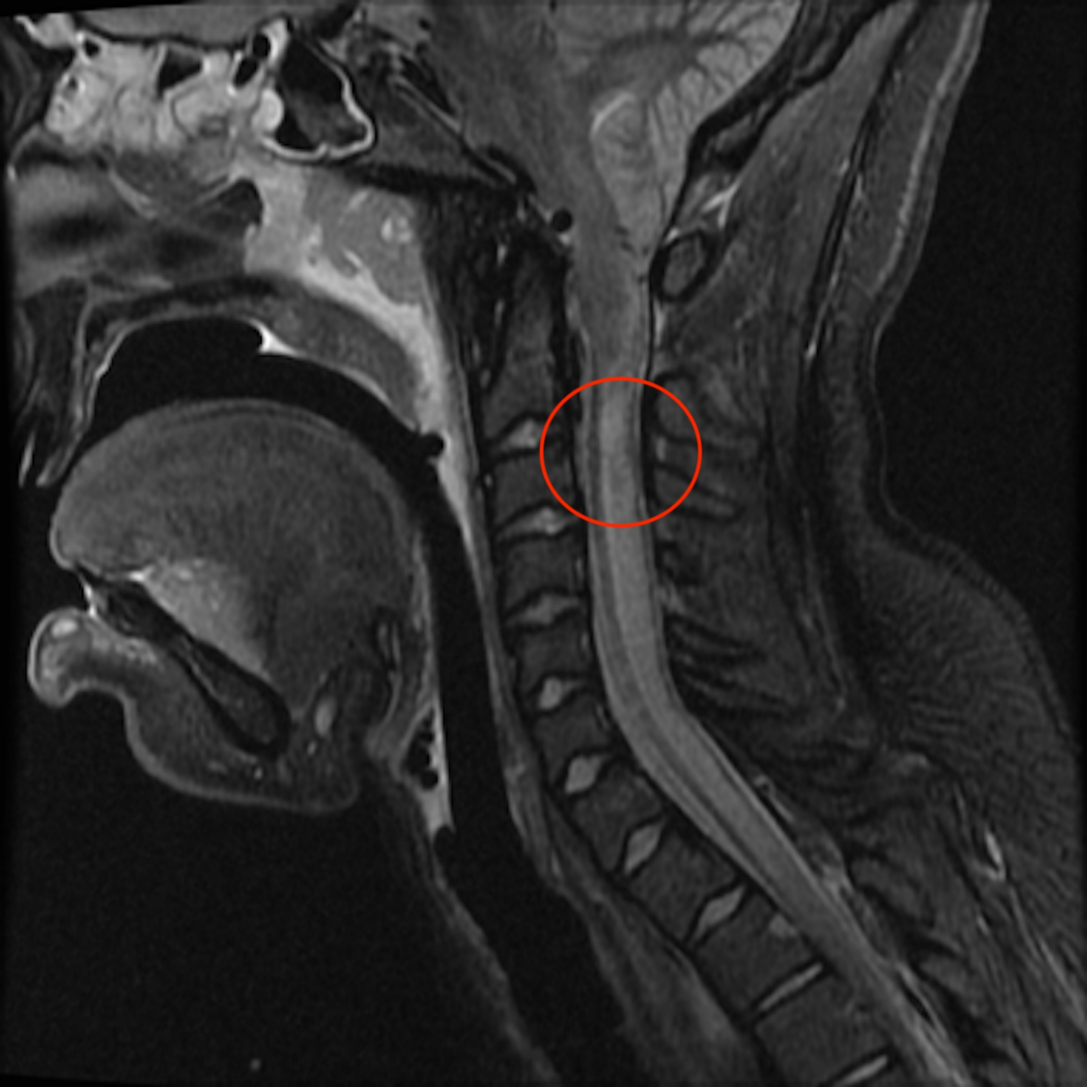
Magnetic resonance imaging (MRI). MRI brain showing Chiari I malformation along with syrinx at C4 and presyrinx down to T2 along with cervical spine venous engorgement and cerebrospinal fluid (CSF) flow obstruction at level of foramen magnum.

An 18-year-old female status post normal vaginal delivery with epidural analgesia two months prior presented to the emergency department (ED) with intermittent headaches, diplopia, nausea/vomiting and cachexia since given birth. She denied prior history of headache. Shortly after arrival to the ED, she became unresponsive and developed flaccid quadriparesis with complete ophthalmoplegia. She was unable to protect her airway and required intubation. Computed tomography (CT) head showed complete basal cistern effacement with 2 cm cerebellar tonsillar herniation into the foramen magnum. Raising the head of the bed caused acute bradycardia and hypotension. She was subsequently given mannitol and required prolonged Trendelenburg positioning to maintain a goal mean arterial pressure (MAP) of 80-100 mmHg. She was not a candidate for decompressive surgery given her hemodynamic and neurologic instability. Magnetic resonance imaging (MRI) of the brain and spinal cord revealed a syrinx at C4 and pre-syrinx extending down to T2 along with cervical spine venous engorgement and cerebrospinal fluid (CSF) flow obstruction at the level of the foramen magnum (Figure 1).

**Figure 2:**
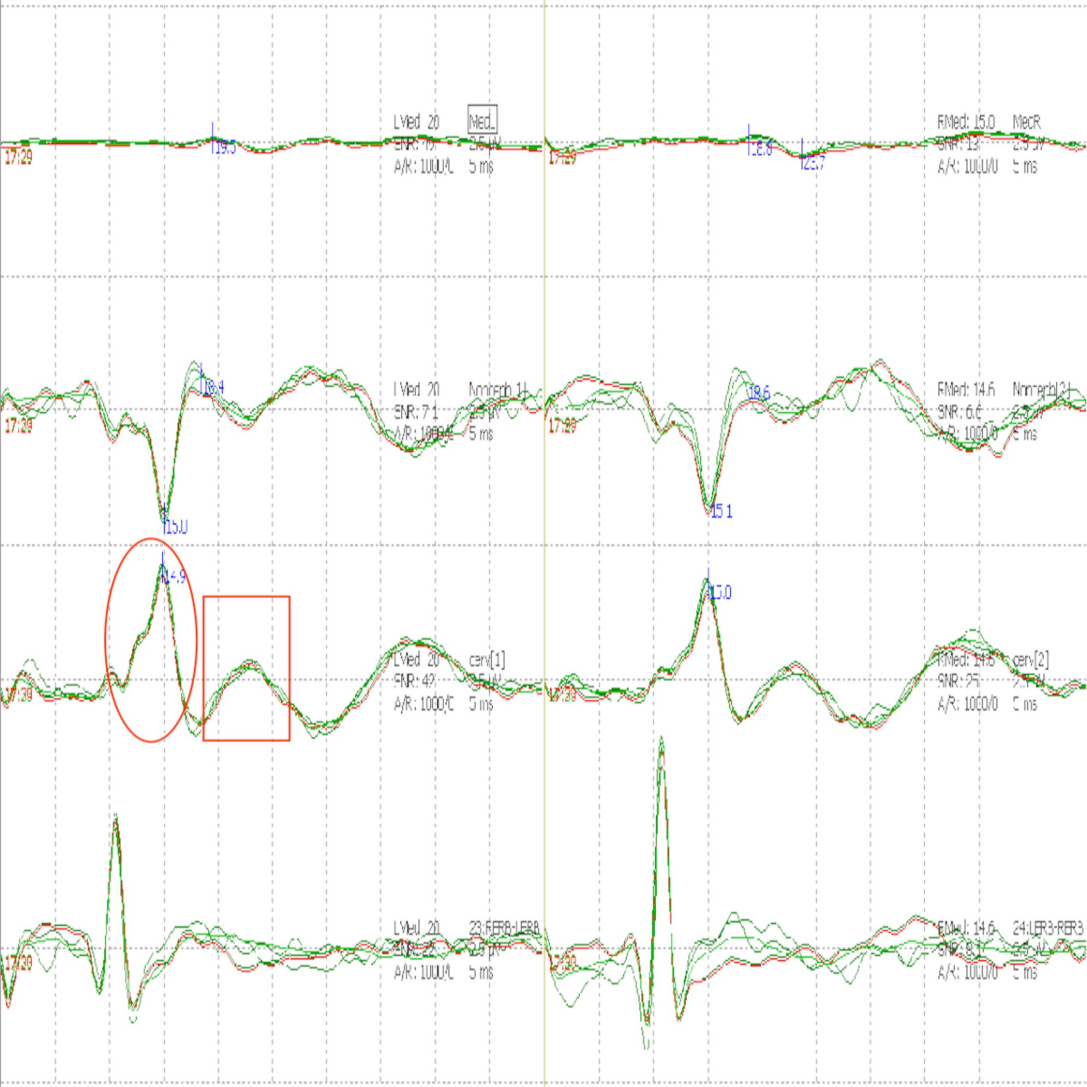
Somatosensory evoked potential (SSEP). SSEP showing decreased N20 amplitude bilaterally (boxes) along with preserved N13 responses (circles).

She was evaluated with serial SSEPs which initially showed low amplitude N20 response bilaterally with preserved N13 responses (Figure 2).

**Figure 3:**
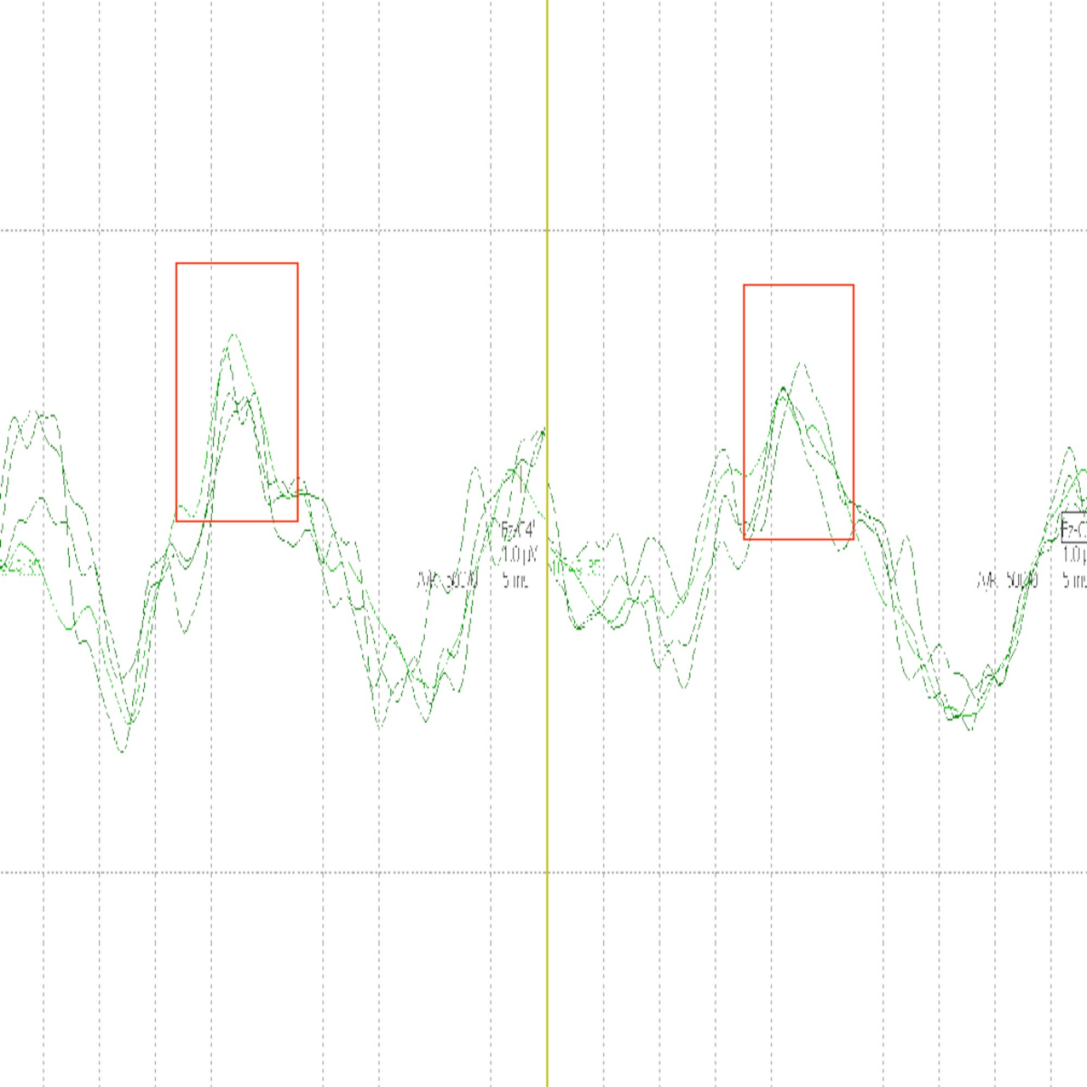
Somatosensory evoked potential (SSEP). SSEP showing increased amplitude of N20 responses bilaterally (boxes).

SSEPs subsequently showed an increase in N20 amplitude (Figure 3) at which point the decision was made to raise the head of bed slowly.

**Figure 4:**
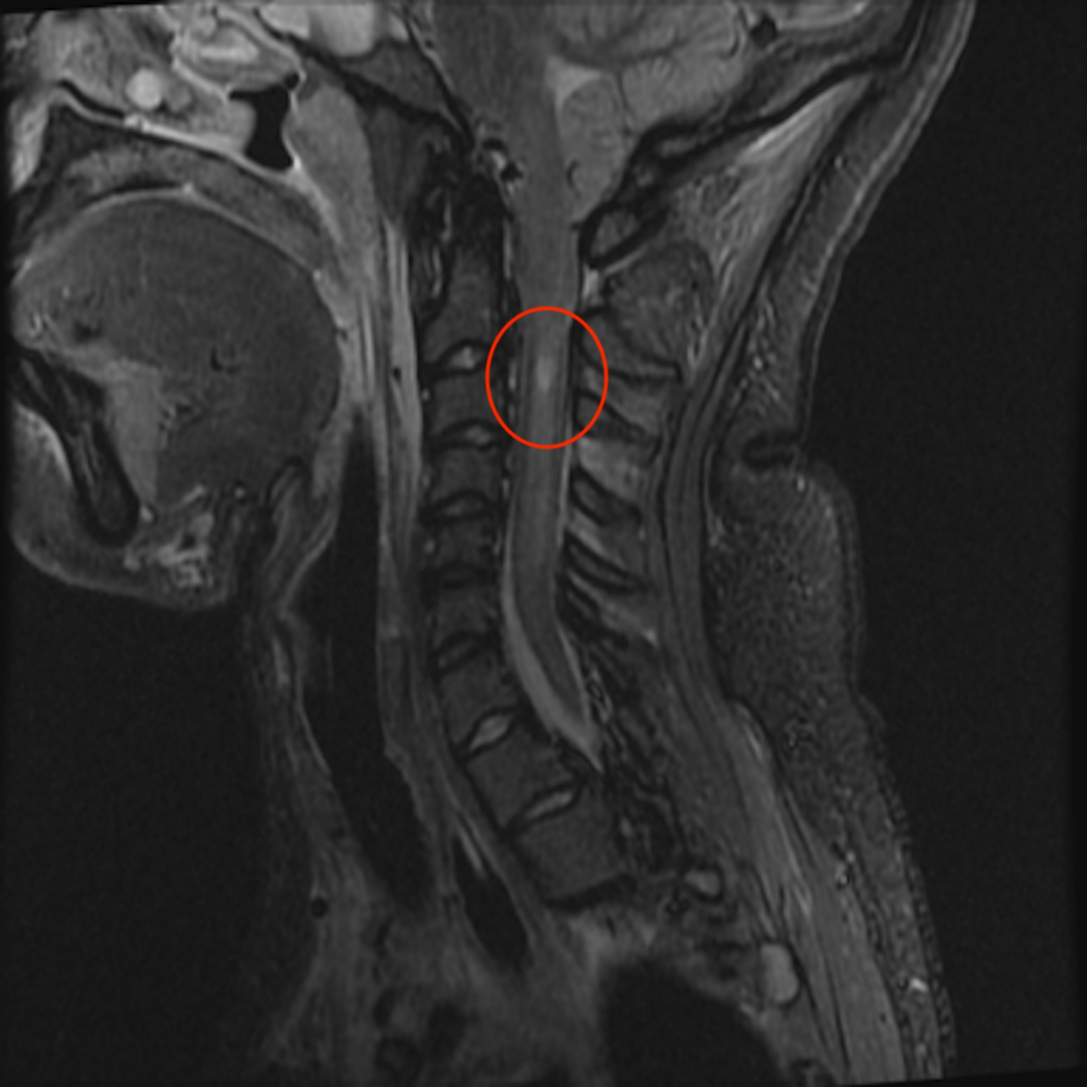
Magnetic resonance imaging (MRI). MRI brain showing resolved presyrinx along with cerebrospinal fluid (CSF) in premedullary region.

During this process, dexmedetomidine, midodrine, and fludrocortisone were used to maintain autonomic stability. Following clinical stability, she was able to tolerate repeat MRI brain (Figure 4) which revealed CSF in pre-medullary regions not evident on initial MRI.

## Discussion

This case highlights the utility of SSEPs in monitoring a patient in the neurological intensive care unit with brainstem compression and a limited clinical exam. Low amplitude median N20 responses were noticed which subsequently improved along with the clinical condition of the patient. The N20 response is a scalp potential with a latency of 20 ms which reflects postsynaptic potentials produced by neurons in the primary somatosensory cortex in response to an afferent thalamocortical electrical impulse [5]. Abnormal N20 amplitude correlated with thalamocortical dysfunction in this patient with a consistently preserved N13 response indicating normal lower cervical cord/cervicomedullary gray matter function despite the presence of syrinx. The role of SSEP in evaluating posterior fossa lesions was highlighted by Shimbo et al. They studied the relationship between location of brainstem lesions detected by MRI and abnormality of brainstem auditory evoked potentials (BAEPs) and short-latency SSEPs in 57 patients with posterior fossa stroke; they found that abnormal SSEPs were associated with lesions of the pontine tegmentum [6]. Recognizing the sensitivity of SSEP to posterior fossa lesions, their role in the NICU is promising. Posterior fossa mass lesions can affect vital brainstem functions which may present as a rapid decline in mental status. SSEP may have a clinical role in assessing worsening or improving brainstem dysfunction in posterior fossa lesions. One study reported the usefulness of SSEP in predicting repeatability of brainstem function following infratentorial epidural brain compression in animal models. This study described SSEP as a more sensitive early predictor of deteriorating neurological function than neurological examination and reported that the SSEPs can be useful in predicting electrophysiological repeatability [7]. Given the limitations of CT in assessing the posterior fossa, SSEPs may be a useful monitoring adjunct to assess the evolution of posterior fossa lesions in patients who cannot obtain MRI imaging due to clinical instability.

Our patient presented with complaints of intermittent headache and diplopia and subsequently developed flaccid quadriparesis with complete ophthalmoplegia. CT head confirmed the clinical hypothesis of intracranial hypotension and showed complete cisternal effacement in the setting of, what we suspect, was a pre-existing Chiari 1 malformation causing cerebellar tonsillar herniation into the foramen magnum that worsened following epidural analgesia. We suspect the intracranial hypotension was secondary to CSF leak following dural trauma from epidural spinal anesthesia performed two months earlier. CSF exerts necessary buoyant force on cranial contents, protecting them against downward traction. Intracranial hypotension decreases buoyancy, thus predisposing to brainstem compression. Ophthalmoplegia in this patient may have resulted from pontine flattening against the clivus leading to cranial nerve compression. However, because transient ischemia in the acute phase of intracranial hypotension can also result in cranial nerve paresis, we targeted a higher MAP. Although not observed in this patient, tonsillar herniation can result in downbeat or gaze-evoked nystagmus [8]. Visual acuity and field deficits may also occur as a result of stretching of the optic complex over the pituitary fossa, or secondary to vascular congestion of the optic nerves and tracts [9]. The exact mechanism of cranial nerve paresis in intracranial hypotension is unclear. In the majority of cases, ocular findings reverse with correction of intracranial hypotension. Patients with intracranial hypotension typically respond favorably to conservative treatment such as recumbent positioning, intravenous fluids, and intravenous caffeine administration. Epidural blood patch can be used for refractory intracranial hypotension if conservative measures fail [9,10].

Our patient required a prolonged course of Trandelenburg position given her hemodynamic instability. She was monitored with serial SSEP. Once clinical and electrophysiologic improvement was noted, the head of the bed was slowly raised. Dexmedetomidine, midodrine, and fludrocortisone were used to treat dysautonomia. Normalizing SSEPs correlated with clinical and radiographic improvement.

## Conclusions

Our study demonstrates the utility of SSEPs in monitoring a patient with a cervicomedullary compression from intracranial hypotension. Given the sensitivity of SSEPs to evaluate dorsal column and medial lemniscus somatosensory pathways, it may be useful in monitoring the evolution of posterior fossa lesions.
